# Demographic and clinical parameters are comparable across different types of pediatric feeding disorder

**DOI:** 10.1038/s41598-022-12562-1

**Published:** 2022-05-21

**Authors:** Tut Galai, Gal Friedman, Michal Moses, Kim Shemer, Dana L. Gal, Anat Yerushalmy-Feler, Ronit Lubetzky, Shlomi Cohen, Hadar Moran-Lev

**Affiliations:** 1 Department of Pediatric Gastroenterology, Dana Dwek Children’s Hospital, Tel Aviv Medical Center, Tel Aviv, Israel; 2 Department of Pediatrics, Dana Dwek Children’s Hospital, Tel Aviv Medical Center, Tel Aviv, Israel; 3 Sackler Faculty of Medicine, Tel Aviv University, Tel Aviv, Israel

**Keywords:** Nutrition disorders, Risk factors

## Abstract

Knowledge and understanding of risk mechanisms associated with pediatric feeding disorder (PFD) remain limited. We aimed to investigate factors associated with PFD and their relation to specific PFD types according to the recent consensus WHO-based definition. We retrospectively reviewed the medical records of children with PFD and retrieved their demographic and clinical characteristics. Healthy age- and sex-matched children served as controls. Included were 254 children with PFD [median (interquartile range) age 16.4 (9.5–33) months at diagnosis] and 108 children in the control group [median age 24.85 (14.5–28.5) months]. According to the WHO-based definition, disturbances in oral intake were predominantly related to nutritional dysfunction in 118 (46.6%), feeding skill dysfunction in 83 (32.3%), medical conditions in 42 (16.7%) and psychosocial dysfunction in 11 (4.4%). In multivariate analysis, children with PFD had a higher risk for lower socioeconomic background (*P* < 0.01) and low birth weight (26.8% compared to 7.4%, *P* < 0.001). Moreover, significantly fewer children in the PFD group were breastfed (75% versus 89%, *P* = 0.003). There were no significant differences in any of those variables between PFD types. In conclusion, low socioeconomic status, lack of breastfeeding, and low birth weight were significantly more frequent in children with PFD. PDF manifest as multiple dysfunctions, thus highlighting the need to offer these children and their families multidisciplinary care.

## Introduction

Feeding disorders in the pediatric population comprise a heterogeneous group of conditions with no universally accepted classification. They may include, but are not limited to, poor appetite, food selectivity, food refusal, and delayed or absent development of feeding skills which may or may not be accompanied by inappropriate growth^1^. Feeding disorders are common in the pediatric population, with a prevalence of 25% of children in the general population and of up to 80% of children with developmental disabilities^2^. They are reported to be a cause of concern in up to 20% of parents, resulting in considerable numbers who search professional help^3^.

A unifying diagnostic definition of pediatric feeding disorder (PFD) was proposed by a panel of experts well versed in the care of children with feeding disorders^4^. Their proposed consensus definition and conceptual framework was based upon the framework of the World Health Organization (WHO) International Classification of Functioning (ICF), Disability and Health^5^, and was accepted as part of the ICD diagnostic nomenclature as of October 2021 (ICD-10-CM R63.31). The World Health Organization ICF framework defines functioning as an umbrella term referring to all body functions, activities, and participation, and defines disability as an umbrella term covering impairment (a problem in body function or structure), activity limitation (difficulty encountered in executing a task or action), and participation restriction (problem experienced in involvement in life situations)^5^. According to the new definition, PFD comprise impaired oral intake that is not age-appropriate and that is associated with medical, nutritional, feeding skill, and/or psychosocial dysfunction. This classification system describes the impact of PFD on a child’s physical, social, emotional, and cognitive functions, as well as on the caregiver-child relationship, and allows better characterization of heterogeneous populations in order to include all relevant disciplines in the treatment protocol^4^.

Feeding disorders in the pediatric population had been historically divided between organic and non-organic based on medical or psychological/behavioral problems, respectively^6,7^. The International Statistical Classification of Diseases and Related Health Problems, 10th Revision (ICD-10) for PFD requires the absence of organic disease^8^. The Diagnostic and Statistical Manual of Mental Disorders, 5^th^ (DSM-V) Edition diagnosis of avoidant/restrictive food intake disorder incorporates nutritional complications and acknowledges that feeding disorders can interfere with psychosocial functioning and are also common in certain medical conditions^9^. The DSM-V diagnosis, however, requires that the priority of the eating disturbance exceeds that associated with the underlying condition and specifically excludes children whose primary challenge is a feeding skill deficit. Since the etiology of PFD is often multifactorial and involves the disruption of more than one system, a multidisciplinary approach to diagnosis and intervention appears to be optimal for better identifying and treating the underlying causes^10^.

Early prediction of a PFD is an important goal in order to facilitate diagnosis and appropriate treatment. Previous studies identified obstetric factors (such as preterm birth and low birth weight (LBW)), and maternal factors (such as anxiety, depression, eating disorders, and a problematic mother–child relationship)^11–13^ that are associated with childhood feeding problems^14–17^. However, the feeding disorder in previous studies was defined inconsistently with no unified all-inclusive definition. Therefore, the aim of this study was to assess predictive factors for PFD and their relation to a specific feeding disorder type according to the recent WHO-based definition.

## Materials and methods

### Patient population and study design

This retrospective study of infants and children with PFD was based upon data collected from medical records of patients treated in the multidisciplinary feeding clinic of the Institute of Pediatric Gastroenterology, Hepatology and Nutrition at "Dana-Dwek" Children's Hospital, Tel Aviv Medical Center, from January 2018 to January 2020. Included in the study were infants and children aged 0 to 6 years who were referred to the feeding disorder clinic with a clinical diagnosis of PFD^9^. Excluded were 56 subjects who were seen at the clinic in whom the diagnosis of a PFD was ruled out according to the new consensus definition (see below) and 36 patients with missing data. A control group of 108 healthy children was recruited from the general population, and their parents were reached through social media networks and completed a structured questionnaire consisting of clinical and sociodemographic data identical to the data collected for the study group.

The study protocol was approved by the “Helsinki” institutional review board of the medical center. reference number—TLV-0590–20. Informed consent of the participants was waived by the Helsinki institutional review board of the medical center, since the data retrieved from the medical records were anonymous to the researchers​. The data were handled in accordance with the Principles of Good Clinical Practice. All methods were performed in accordance with relevant guidelines and regulations.

### Data collection

The Feeding Disorder Clinic team includes a gastroenterologist, dietitian, speech therapists, and a psychologist. The information in the medical files contains both self-reported patient/parental information and the team’s notes on diagnoses, management, and surveillance. All hospital medical records are electronic, with additional access to the individual's health maintenance organization laboratory data. The information retrieved from medical files of subjects included:

1. Sociodemographic characteristics: age, sex, home address, country of birth, number of children in the family, parents’ occupations, parental marital status.
2. Medical history: perinatal characteristics (pregnancy complications, delivery method, birth weight, gestational age, birth complications), medications, developmental status, background conditions, and hospitalizations.
3. Feeding history: feeding method-breastfeeding or bottle-feeding, age at introduction of complementary food.

## Definition of study variables

Socioeconomic status (SES) was determined by the patient’s home address according to the Israel Central Bureau of Statistics’ Characterization and Classification of Statistical Areas within Municipalities and Local Councils by the Socio-Economic Level of the Population 2015^17^. The SES was scored by clusters of localities of residence, ranging from 1 to 10, with 1 being the lowest rating and 10 the highest. The SES index is an adjusted calculation of 14 variables that measure social and economic levels in the domains of demographics, education, standard of living, and employment (ranging from the lowest [− 2.797] to the highest [2.590]). LBW was defined as a birth weight (BW) below 2500 g. Pregnancy complication was defined as any high-risk pregnancy due to maternal or fetal problems (e.g., gestational diabetes mellitus, preeclampsia, cholestasis, intrauterine growth retardation [IUGR], multiple pregnancy).

PFD was diagnosed according to the new ICD diagnostic term as a disturbance in oral intake of nutrients inappropriate for age lasting at least 2 weeks and an absence of cognitive processes consistent with eating disorders and pattern of oral intake not due to a lack of food or to being incongruent with cultural norms^4^.

The type of feeding disorder had been defined by the clinical team based upon the anamnesis of the patients and parents and reports in the medical files. The same team retrospectively reviewed the medical files and, based upon that information, divided the types into a nutritional disorder (any case of malnutrition, specific nutrient deficiency, or reliance on oral supplements to sustain nutrition), feeding skill dysfunction (use of modified feeding strategies, position, or food texture), medical (any medical conditions that interfere with normal age-appropriate eating practice, e.g., cleft palate, absent swallowing reflex, etc.), and psychosocial dysfunction (any case of avoidance behaviors when being fed or inappropriate caregiver management of the child’s feeding). The code of the predominant type was used in the event of overlap between PFD manifestations. There was disagreement between the team in fewer than 10% of the cases, and the dominant type was the one that received the most votes.

### Statistical analyses

The data were analyzed with the Statistical Package for the Social Sciences software version 27 (SPSS Inc., Chicago, IL). All statistical tests were 2-sided. The Kolmogorov–Smirnov test and the Shapiro–Wilk test were applied to assess the normality of continuous data. The data are expressed as means ± standard deviation (SD) for normally distributed variables and median and interquartile range (IQR) for skewed distribution. Pearson's chi-square test or Fisher's exact test was performed to compare the distribution of categorical variables between children with PFD and controls. An independent sample t-test or an independent sample Mann–Whitney was performed to compare between groups for continuous variables with a normal or skewed distribution, as appropriate. The Kruskal Wallis test was used to compare 4 types of PFD. Multivariate logistic regression models were used to assess the association between PFD and associated factors. A *P* value < 0.05 was considered significant.

### Ethics approval and consent to participate

The study protocol was approved by the “Helsinki” institutional review board of the medical center. reference number—TLV-0590–20. Informed consent of the participants was waived by the Helsinki institutional review board of the medical center, since the data retrieved from the medical records were anonymous to the researchers.

## Results

### Study population

**Figure 1:**
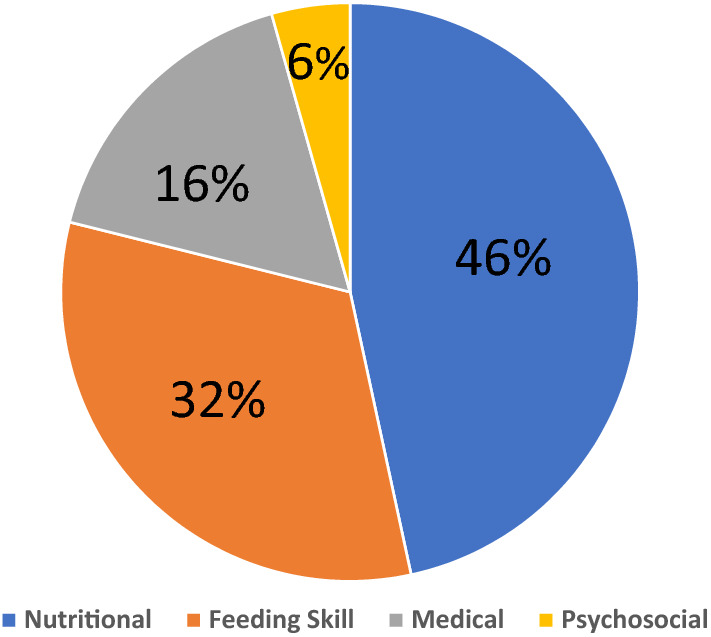
Pediatric feeding disorder types according to the recent consensus definition.

Table 1 lists the demographic and clinical data of the children with PFD. The cohort of children and infants with PFD was comprised of 254 patients, 142 (56.1%) males, and a median age of 16.34 (IQR 9.5–33) months. Among them, 121 children (47.9%) were referred to the clinic by their pediatrician, 38 (15.1%) were referred after evaluation by specialist physicians (neurologists or gastroenterologists), 18 children (7%) were referred after hospitalization, and 77 children (30%) were self-referrals. According to the recent consensus PFD definition, the disturbance in oral intake was predominantly related to a nutritional dysfunction in 118 children (46.6%), to feeding skill dysfunction in 83 children (32.3%), to a medical condition in 42 children (16.7%) and to psychological dysfunction in 11 children (4.4%, Fig. 1). Eighteen children (7%) were treated with cyproheptadine, and 40 children (16%) were treated with proton pump inhibitors. Twelve (4.7%) children received nutritional supplementation via gastrostomy. The age- and sex-matched control group was comprised of 108 children, 56 (51.9%) males, median age 24.85 (interquartile range [IQR] 14.5–28.5) months.

**Table 1:** Demographic and clinical characteristics of children with feeding disorders compared to controls.

	Feeding disorder group *n* = 254	Control group *n* = 108	*P*
Sex (male)	142 (56.1)	56 (51.9)	NS
Age, months	16.4 (9.5,33)	24.85 (14.5,28.5)	NS
**Socioeconomic position**			
Cluster (mean ± SD)	6.71 ± 1.93	7.63 ± 1.07	0.001
Index (mean ± SD)	1.02 ± 0.41	0.7 ± 0.8	0.01
**Parents’ marital status**			
Married	192 (75.9)	101 (93.3)	
Divorced	17 (6.9)	3 (3.3)	0.004
Single parent	44 (17.2)	3 (3.3)	
Number of siblings	1.53 ± 1.32	0.87 ± .87	< 0.001
Parental feeding disorder	108 (42.7)	8.31 (7.7)	< 0.001
Multiple pregnancy	24 (9.5)	3 (2.8)	0.02
IUGR	47 (18.8)	1 (0.9)	< 0.001
Low birth weight	68 (26.8)	8 (7.4)	< 0.001
Delivery by C/S	96 (37.8)	22 (20.4)	0.001
Preterm	52 (20.5)	6 (5.6)	< 0.001
Birth weight, grams	2992 (2489, 3363)	3255 (2938, 3600)	< 0.001
Breastfeeding	189 (74.8)	102 (88.8)	0.003
Age of complementary food introduction, month	6 (4, 6)	6 (5, 6)	NS
Developmental delay	91 (35.8)	9 (8.3)	< 0.001
Medication	68 (26.9)	6 (5.6)	< 0.001
Hospitalization	73 (29)	15 (13.9)	0.002
Background disease	94 (37.2)	21 (19.4)	0.001
Allergy	21 (8.3)	6 (5.6)	NS

The data are expressed as median and interquartile range for continuous variables, and *n* (%) for categorical variables.

*IUGR* intrauterine growth restriction, *C/S* cesarean section.

### Factors associated with PFD

A comparison between the children diagnosed as having a PFD and the control group revealed significant clinical differences. More children in the former group were born preterm or were one of twins compared to the control group (9.5% and 20.5% versus 2.8% and 5.6%, respectively, Table 1). Moreover, more children in the PFD group had been diagnosed with IUGR or were LBW, with a significant lower mean BW (18.8% were IUGR, 26.8% were LBW, with a mean BW of 2992 g in the PFD group compared to 0.9% IUGR and 7.4% LBW with a mean BW of 3255 g in the control group, *P* < 0.001). The odds ratio (OR) and 95% confidence interval (CI) for preterm delivery were 4.379 (1.818–10.546) and 3.668 (1.081–12.453) for multiple pregnancy in the PFD group.

There were also sociodemographic differences between the study and control groups. The families of the children with PFD had a significantly lower SES based on cluster and index scores (mean SES cluster 6.71 ± 1.93 and mean index 1.02 ± 0.41 for the PFD group compared to 7.63 ± 1.07 and 1.2 ± 0.41 in the control group, *P* < 0.01). Moreover, the numbers of divorced or single parents were higher in families of children with PFD compared to controls (14.1% vs 6.6%, respectively, *P* = 0.004), and the total number of siblings was higher (1.53 ± 1.3 vs. 0.87 ± 0.87, respectively, *P* < 0.001).

Significant differences were also noted during the neonatal period. Significantly fewer children in the PFD group were breastfed compared to the control group (75% vs. 89%, respectively, *P* = 0.003). The children with PFD had significantly more background conditions (such as epilepsy, asthma, laryngomalacia, etc.) (37.2% vs. 19.4%, *P* = 0.001), and hospitalizations (29% vs 13.9%, *P* = 0.002), and were treated more with prescription medications (26.9% vs 5.6%, *P* < 0.001) compared to the controls. There was no group difference in the age of complementary food introduction or in documented allergy to food.

Stepwise backward logistic regression, in which the PFD was the dependent variable and sex, multiple pregnancies, BW, preterm delivery, breastfeeding, and SES (examined by SES cluster) were independent variables, demonstrated that the rates of a low SES, lack of breastfeeding, and LBW were significantly higher in children with PFD compared to controls (Table 2). The OR (95% CI) for low BW among children with PFD was 3.75 (1.67–8.42), for low SES 0.64 (0.51–0.81) and for no breastfeeding was 0.41 (0.19–0.89).

**Table 2:** Multivariate logistic regression for a feeding disorder.

Variable	Odds ratio	Lower 95% CI	Upper 95% CI	*P*
Breastfeeding	0.41	0.19	0.89	0.02
SES cluster	0.64	0.51	0.81	< 0.001
Low birth weight	3.75	1.67	8.42	0.001

*CI* confidence interval, *SES* socioeconomic Status.

### Factors associated with PFD according to PFD type

Table 3 lists factors associated with PFD according to PFD type. There was no difference in sex distribution, gestational age, or rates of multiple pregnancies, preterm deliveries, or IUGR between the study and control groups. There were fewer cesarean section deliveries in the psychosocial PFD group type compared to the 3 other group types. As expected, there were significant differences in the number of prescribed medications, developmental delays, and prior hospitalizations between the medical PFD group compared to all other groups. Finally, no group differences were noted in the socioeconomic variable (cluster, index, marital status), breastfeeding rates, and age at introduction of complementary foods.

**Table 3:** Factors associated with feeding disorders according to feeding disorder type.

	Medical *n* = 42	Nutritional *n* = 118	Feeding skills *n* = 83	Psychosocial *n* = 11	*P* value
Sex (male)	19 (13.5)	73 (51.4)	42 (29.5)	8 (5.6)	NS
Gestational age, week	38.5 (36.6,40)	38.26 (38, 40)	39 ^38,40^	38.33 (38.40.5)	NS
Birth weight, grams	2760 (2260,3042.5)	3000 (2500,3300)	3070 (2460,3500)	3045 (2825, 3585)	NS
Multiple pregnancy, *n* (%)	2 (8.3)	11 (45.8)	10 (41.7)	1 (4.2)	NS
IUGR- *n* (%)	11 (23.4)	24 (51.1)	12 (25.5)	0 (0)	NS
Delivery by C/S, *n* (%)	13 (13.3)	49 (51.1)	23 (24.4)	10 (11.1)	0.02*
Preterm, *n* (%)	11 (20.4)	21 (42.9)	17 (34.7)	2 (2)	NS
**Parents’ marital status, *n* (%)**					
Married	15 (22.7)	31 (47)	19 (28.8)	1 (1.5)	NS
Divorced/single parent	8 (9.8)	37 (60.6)	15 (24.7)	3 (4.9)	
SES cluster	7 (5, 8)	7 (7, 8)	7 (7, 8)	8 (7, 8)	NS
SES index	0.8 (− 0.4 to 1.2)	1.05 (0.5,1.2)	1.17 (0.52,1.2)	1.05 (0.69,1.29)	NS
Breastfeeding, *n* (%)	24 (12.2)	93 (49.2)	63(33.6)	10 (5)	0.058
Age of complementary food introduction, month	6 (6,9.5)	5 (4, 6)	6(4, 6)	4.5 (4, 5)	NS
Developmental delay, *n* (%)	32 (35)	35 (38.8)	24 (26.3)	0 (0)	< 0.001**
Medication	20 (29.4)	22 (32.4)	20 (29.4)	6 (8.8)	< 0.001**
Hospitalization	25 (33.8)	24 (32.4)	19 (26.8)	5 (7)	< 0.001**

The data are expressed as median and interquartile range for continuous variables and *n* (%) for categorical variables.

*IUGR* intrauterine growth restriction, *C/S* cesarean section, *SEP* socioeconomic position.

*Difference between psychosocial dysfunction and three other groups.

**Difference between medical dysfunction and three other groups.

## Discussion

We investigated the application of the new diagnostic term, ‘‘pediatric feeding disorder’’, for unifying the medical, nutritional, feeding skills, and psychological concerns associated with PFD. Our results demonstrated that sociodemographic and perinatal factors associated with PFD were similar among all of the selected feeding disorder types, supporting their derivation from a single source and the suitability of this new terminology for diagnosing such disorders in children.

The present study included children with different types of feeding disorder according to the recent consensus PFD definition. The children were diagnosed and followed in a tertiary medical center over the course of 2 years. We compared this cohort to a control group of age- and sex-matched apparently healthy children from the general population. We demonstrated that a low SES, lack of breastfeeding, and a LBW were significant predictors for PFD. We also revealed higher rates of preterm and twin pregnancies, cases of IUGR, divorced and single parents, background conditions, hospitalizations, and prescription medications in the PFD group compared with the control group and that, importantly, these factors were not associated with PFD type.

PFD is a relatively common clinical diagnosis with increasing prevalence, according to a recent study of Kovacic et al.^18^. Very few studies have examined the predictors for PFD, and none of them were related to the specific types of feeding disorder as listed in the recent PFD consensus diagnosis definitions. This new definition provides a consistent terminology, encompassing all feeding disorder domains and disciplines, and recognizing specific subtypes with treatment and prognostic implications^9^.

Our findings revealed several significant predictors for PFD. A low SES as a predictor for PFD is in line with the study of Carpnell et al. who demonstrated that a lower SES is associated with feeding disorders at age 2 years in preterm babies^19^. The development of healthy eating behaviors depends, among other factors, on responsive parenting behaviors, which reflect reciprocity between the child and caregiver, and includes recognition of internal signals of hunger and satiety with a developmentally appropriate supportive response^20^. Caregivers of a lower SES must rely upon multiple caregivers of their children while they work. Moreover, many low-wage jobs do not support breastfeeding and therefore may disrupt the child’s acquisition of proper eating habits. In addition, they have lower economic and social resources ^20^, which may limit their ability to offer diverse and nutritious food to their children.

We found a higher prevalence of LBW, preterm births, and twin pregnancies in the study group compared to the control group. Few studies have demonstrated that infants born preterm are at high risk of oral feeding difficulties during the neonatal period^21,22^ and throughout childhood^23,24^. This could be associated with prolonged nasogastric feeding and respiratory support, delayed oral feeding^25^, and early hypotonia ^19^. Samara et al. reported that eating problems were more common at 6 years of age among children born extremely preterm (< 32 weeks) compared to a term control group, and that they include oral motor, hypersensitivity, and behavioral problems^23^. Johnson et al. showed that late-moderate preterms^29–32,34^ were at increased risk to develop picky eating habits and oral motor problems at 2 years corrected age compared with their term peers^24^. Unlike those studies, we did not observe any association between a specific type of PFD and being born preterm. Other studies demonstrated more parental stress and concerns among mothers of preterms^27^ and multiples^26^ which led to their difficulty in interpreting infant behaviors and adjusting meals volumes and schedules. The authors concluded that guidance on feeding these infants after discharge from maternity facilities is needed^27^.

Migraine et al.^12^ demonstrated that preterm and low BW children have more eating difficulties than term children at 2 years of age. However, as in our study, after adjustment for maternal and neonatal characteristics, a BW z score <  − 1, but not gestational age, was associated with PFD. This emphasizes that weight probably has a greater influence than gestational age on the development of an eating disorder. Interestingly, those authors observed that a maternal level of education below high school was also associated with PFD, highlighting the importance of identifying this specific population in order to prevent PFD.

We found that fewer children in the PFD group were breastfed compared to our control group. Breastfed infants are reportedly exposed to varying flavors during lactation originating from the maternal diet^29^, unlike formula-fed infants who lack the exposure to flavor variety. Other studies suggest that breastfed infants are initially more accepting of a wider variety and novel foods during the weaning period, and that repeated exposure to a novel food led to greater acceptance among breastfed infants compared to formula-fed infants^27–29^. This effect is still evident at 3 and 6 years of age^30^. Moreover, children who were breastfed appear to eat more fruits and vegetables, be less picky, and show lower levels of neophobia (fear from new foods) during later childhood compared to children who were formula-fed^31,34^.

To the best of our knowledge, this is the first study to provide clinically based evidence on predictors of PFD according to the recent consensus definition. The diagnosis of PFD was made by a multidisciplinary team in a clinical setting. This is also the first description of the PFD subtypes included in the new ICD diagnostic term. The findings in our study that sociodemographic and perinatal factors associated with PFD are similar among PFD types may support the notion that these types derive from a single source and strengthen the appropriateness of the usage of this new terminology for PFD diagnosis. Their manifestations as medical, nutritional, feeding, and psychological dysfunctions highlight the need to offer these children and their families multidisciplinary care in order to address all domains that may be involved. Early diagnosis of children at risk for PFD and appropriate multidisciplinary care can improve the prognosis of these children and allow them to develop age-appropriate eating habits.

This observational study is limited by its retrospective nature and by the absence of more precise data on growth parameters and the mental status of the children and parents. Some degree of incompatibility between clinical diagnosis and the new formal PFD definition may be anticipated. However, since the diagnosis of PFD was made by a multidisciplinary team comprised of a pediatric gastroenterologist, dietician, speech therapist, and psychologist, taken together with the anonymity of the parental questionnaire, we believe that inaccuracies in PFD and its subtypes were held to a minimum. Moreover, the absence of an eating disorder in the control group was determined by their parents and may have included children whose eating habits were actually not appropriate. Lastly, the patient cohort accessed care at a subspecialty clinic and they may not represent the broader pediatric population. However, our hospital is a tertiary care hospital which we believe does represent the general pediatric population.

In conclusion, we presented sociodemographic and perinatal factors associated with PFD and demonstrated that these factors are not affected by the PFD subtype, supporting the usage of the unifying diagnostic classification term and the need for multidisciplinary management. Further studies should investigate the effect of different approaches for treating PFD subtypes.

## Data Availability

The datasets used and/or analyzed during the current study are available from the corresponding author on reasonable request.

## Abbreviations

PFD: Pediatric feeding disorder
ICD-10: International Statistical Classification of Diseases and Related Health Problems, 10th Revision
DSM-V: The Diagnostic and Statistical Manual of Mental Disorders
WHO: World Health Organization
LBW: Low birth weight
SEP: Socioeconomic position
IUGR: Intrauterine growth retardation [IUGR]
BW: Birth weight
IQR: Interquartile range
C/S: Cesarean section
